# Executive Function-Related Improvements on a Commercial CBT-Based Weight Management Intervention: Pilot Randomized Controlled Trial

**DOI:** 10.3390/ijerph19148763

**Published:** 2022-07-19

**Authors:** Andreas Michaelides, Ellen Siobhan Mitchell, Heather Behr, Annabell Suh Ho, Grant Hanada, Jihye Lee, Sue McPartland

**Affiliations:** 1 Academic Research, Noom, 229 W 28th St., New York, NY 10001, USA; esiobhanmitchell@gmail.com (E.S.M.); hbehr@saybrook.edu (H.B.); annabell@noom.com (A.S.H.); 2 Department of Integrative Health, Saybrook University, 55 W Eureka St., Pasadena, CA 91103, USA; 3 Intheon Labs, 5355 Mira Sorrento Pl, Suite 260, San Diego, CA 92121, USA; grant.hanada@intheon.io; 4 Department of Communication, Stanford University, 450 Jane Stanford Way, Stanford, CA 94305, USA; jihyelee@stanford.edu; 5 Research Department, Focus Feedback LLC, 358 Veterans Memorial Highway, Commack, NY 11725, USA; suemcpartland@gmail.com

**Keywords:** CBT, obesity, mobile health, executive function, EEG

## Abstract

Executive functioning is a key component involved in many of the processes necessary for effective weight management behavior change (e.g., setting goals). Cognitive behavioral therapy (CBT) and third-wave CBT (e.g., mindfulness) are considered first-line treatments for obesity, but it is unknown to what extent they can improve or sustain executive functioning in a generalized weight management intervention. This pilot randomized controlled trial examined if a CBT-based generalized weight management intervention would affect executive functioning and executive function-related brain activity in individuals with obesity or overweight. Participants were randomized to an intervention condition (N = 24) that received the Noom Weight program or to a control group (N = 26) receiving weekly educational newsletters. EEG measurements were taken during Flanker, Stroop, and N-back tasks at baseline and months 1 through 4. After 4 months, the intervention condition evidenced greater accuracy over time on the Flanker and Stroop tasks and, to a lesser extent, neural markers of executive function compared to the control group. The intervention condition also lost more weight than controls (−7.1 pounds vs. +1.0 pounds). Given mixed evidence on whether weight management interventions, particularly CBT-based weight management interventions, are associated with changes in markers of executive function, this pilot study contributes preliminary evidence that a multicomponent CBT-based weight management intervention (i.e., that which provides both support for weight management and is based on CBT) can help individuals sustain executive function over 4 months compared to controls.

## 1. Introduction

Obesity has become increasingly prevalent worldwide, affecting approximately 38.2% of adults in the US, and increases risk of health complications such as type 2 diabetes and cardiovascular disease [1]. Behavioral lifestyle weight management interventions are effective, first-line treatments for obesity [2]. Research is increasingly discovering that the processes implicated in weight management are not just physical, but also cognitive in nature [3]. For example, executive function is a term that describes the cognitive processing and coordination involved in behavioral change, such as planning or inhibition of complex behaviors and performance monitoring [4]. Executive functions are intimately involved in goal-setting processes, such as focusing effort towards important weight or eating goals, and inhibiting irrelevant behavior or response to stimuli [5,6]. They are also involved in the coordination and monitoring of weight-related behaviors such as eating behaviors or physical activity [6]. Overall, this body of work suggests that executive functioning is relevant to effective weight management.

While standard interventions tend to focus on weight-related behaviors and goals, recent research suggests that improving executive functioning could be beneficial for individuals with obesity. Studies and reviews suggest that impaired executive functioning is associated with difficulties losing or maintaining weight [3,6,7,8]. Further, studies have shown that improvements in executive function are associated with better future weight loss or adherence to healthy behaviors [9,10,11]. This work raises the possibility that improving or maintaining executive function through weight management interventions could improve weight loss success.

There is mixed evidence on whether weight loss, whether done on one’s own or through standard weight management interventions, improves executive function [6,12,13], necessitating more data, especially from randomized trials. Further, there is some evidence that weight management interventions that target cognitive processes related to executive functions could improve executive functioning compared to usual care. A few studies have shown that cognitive remediation therapy for obesity (CRT-O), a type of weight management intervention specifically targeting cognitive processes, may improve executive function [11,14]. Cognitive behavioral therapy (CBT) is one of the most common evidence-based cognitively oriented theoretical frameworks for weight management interventions, and is effective on both in-person and online platforms [15,16,17]. Previous work suggests that CBT as well as third-wave CBT (e.g., mindfulness) may exert effects on executive function by increasing awareness and monitoring of present thoughts and feelings, recognition and response to conflicts between current and ideal thoughts, and modification of thought patterns [18,19]. Randomized trials have shown that mindfulness interventions increase executive functioning and executive functioning-related brain activity compared to controls [18,20,21]. These were not weight management interventions, however, and to our knowledge, it is unknown whether a CBT-based weight management intervention could improve or sustain executive function.

Given the mixed evidence from weight management interventions and the lack of evidence from a CBT weight management intervention, this pilot randomized controlled trial compared executive functioning and neural markers related to executive function in individuals who received a 16-week digital CBT-based weight management intervention (Noom Weight) to a usual care alternative, in this case, a control group who received publicly available digital health and weight loss resources. The executive functions required for self-management, self-monitoring, goal setting, and action planning in weight management are commonly measured by computerized cognitive performance tasks. For example, the Flanker task and the Stroop task measure inhibitory control, or the “voluntary inhibition of dominant or automatic responses for controlling actions, thoughts, and emotions [...] in order to respond appropriately to the needs of goal-directed behaviors”, and the N-back task measures working memory [3] and cognitive workload [22]. Further, brain activity in the form of error-related negativity (ERN) or spectral power are thought to represent executive functioning processes, such as inhibitory control and working memory [23,24]. Those exposed to mindfulness training in the context of mental health show greater ERN magnitude and spectral power than controls [25,26]. Thus, in this pilot study, we hypothesized that the intervention group would show better executive function, as measured by the accuracy and reaction time on the Flanker task, Stroop test, and N-back test, compared to a control group over time. We also hypothesized that the intervention group would show increased ERN magnitude (i.e., more negative peak amplitude) and spectral power during the Flanker, Stroop, and N-back tasks than the control group over time.

## 2. Materials and Methods

### 2.1. Study Procedure

**Figure 1:**
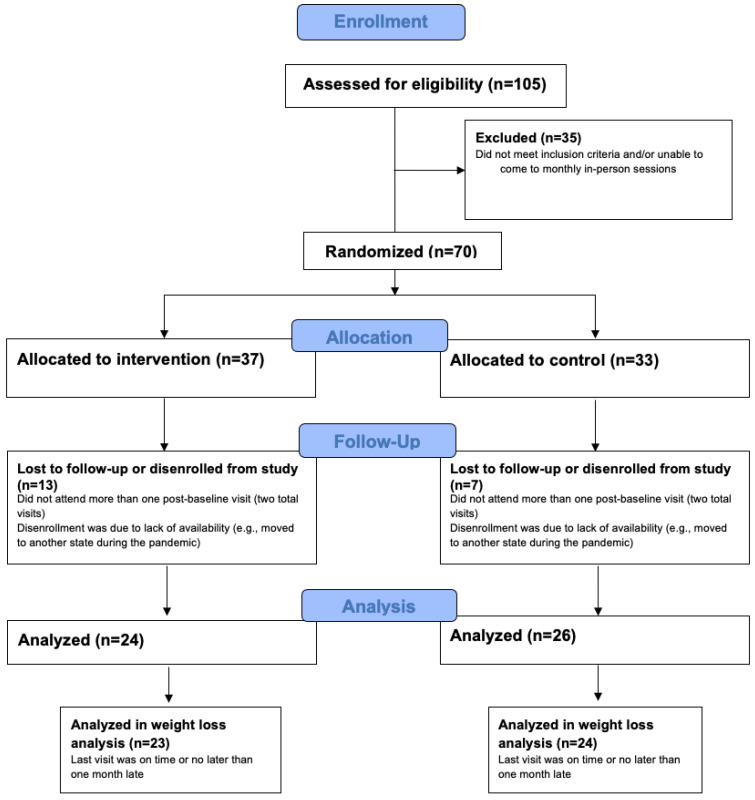
Flow diagram of inclusion and exclusion.

This was a prospective single-center, randomized controlled study. The study was approved by the Advarra IRB. Individuals were recruited from social media and from the signup procedure for Noom Weight and had never used Noom before. They were eligible to participate if they were between 18–60 years old, self-reported good health, had overweight or obesity (BMI ≥ 25), and could attend in-person visits in Long Island, New York. Exclusion criteria were as follows: a visual impairment that cannot be corrected with glasses or contact lenses; indication of drug, alcohol, or medication abuse; history of a brain injury, seizure disorders, or other neurological conditions; presence of an eating disorder or psychiatric disorder, current pregnancy or less than or equal to 6 months postpartum, and use of prescription medications known to impact weight (e.g., weight loss medications). These criteria were assessed via self-report (e.g., “Have you ever suffered from brain injury, seizure disorders, or other neurological conditions?”) in a telephone screening questionnaire. Once eligible and after providing written informed consent, participants were randomized to either the intervention condition which received the 16 week Noom Weight program (N = 37), or the control condition (N = 33). The control condition received a weekly email containing a newsletter with information on healthy eating, weight loss, and physical activity. There were five monthly in-office visits to gather cognitive performance and EEG measurements, occurring at baseline (visit 1) and every four weeks (visit 2, visit 3, visit 4), with visit 5 occurring at week 16, just after completing the 16-week Noom Weight program. Participants were excluded from data analyses if they attended only one post-baseline visit, for a total sample of N = 50 (intervention: N = 24; control: N = 26). To accurately measure weight, participants were excluded from weight loss analyses if their last visit was more than one month later than the planned visit date, for a total sample of N = 47 (intervention: N = 23; control: N = 24). See Figure 1 for a diagram of inclusion in analyses.

Recruitment for this study occurred during the initial months of the COVID-19 pandemic. For social distancing purposes, participants had the option to attend the in-person visits in a mobile testing center or in an office location in Long Island. The mobile testing center contained seating areas and equipment necessary to run the study and was driven to participants’ preferred location. Mobile testing centers have previously been used for cognition testing [27,28], and this testing center provided the same study experience as the office visit (e.g., similar distance from computer screen and the same size screen); the primary difference was that the study took place within a large vehicle, though at rest with the engine off. This vehicle could have had more ambient noise than the office, though it was parked in a quiet residential street for the duration of the session. Pre-testing showed adequate EEG data quality in the mobile testing center. Participants were asked to refrain from drinking caffeinated beverages or engaging in vigorous exercise two hours before each visit. To ensure similar eating (i.e., fed) patterns across visits, each visit occurred at the same time of day and participants were asked to eat the same food at the same time before each visit. At the beginning of each visit, with shoes on, participants were weighed on a scale and their height was measured with a stadiometer. Then, the EEG headset was placed on the participant’s head. After a 6 min resting-state, EEG was measured, and three cognition tests were conducted as follows: practice of N-back task (3 min), N-back task (20 min), break (5 min), practice of Stroop task (3 min), Stroop task (15 min), break (5 min), practice of Flanker task (3 min), Flanker task (17 min), followed by another 6 min resting-state block.

### 2.2. Intervention

Noom Weight is a mobile multicomponent behavior change intervention. The intervention provides education and tools surrounding behaviors that have been shown to relate strongly to successful weight management, such as self-monitoring of food or weight, as well as a daily curriculum based on CBT and third-wave CBT techniques. For example, daily interactive articles will define cognitive behavioral therapy and its components (e.g., what are cognitive distortions), and provide practical and applicable examples and activities (e.g., step-by-step identification and reappraisal of a participant’s cognitive distortion). Other articles may describe mindfulness-based approaches and encourage off-line activities such as deep breathing and mindful eating. In addition to the curriculum, individuals gain access to weight and food logging (self-monitoring) features, text message interactions with a human coach, and virtual groups. Coaches are trained in CBT and motivational interviewing techniques and interact with individuals about their weekly progress towards goals. Participants were not given specific minimum engagement requirements (e.g., read an article every day), and no program components were required for participation; the only general requirement was to complete at least one in-app action at least once per week. The control group did not receive Noom Weight and instead received weekly emailed newsletters with general healthy eating and weight loss tips derived from federal resources, such as the USDA’s Dietary Guidelines for Americans. Given the research question evaluating whether a weight loss intervention with CBT would result in executive functioning differences for individuals with obesity or overweight compared to a usual care alternative, the control group was designed to emulate self-directed weight loss, which is estimated to be the most common method of weight loss in the US [29,30], in which individuals are provided or have access to publicly available materials (e.g., by clinicians or through online searches), and encouraged to follow the guidance to lose weight. Resources were provided weekly to plausibly keep participants blind to group assignment (i.e., increase believability that the newsletters were part of the Noom program and not a separate control group without intervention).

### 2.3. Measures

#### 2.3.1. Cognitive Tasks

For all cognitive tasks, the sample size for each session was made up of the number of participants who attended each session. Sample size discrepancies were accounted for in the mixed linear model, in contrast to a traditional repeated measures ANOVA, in which missing sessions would either need to be imputed or entire subjects would be excluded from the analysis [31].

N-back (2-back): The N-back task [32] is a commonly used measure of working memory function [33]. Randomly selected letters were presented one at a time and participants pressed a key based on whether the current letter is the same (“match”) or different (“non-match”) as the N numbered previous cycle. In this study, a 2-back task was used such that the current letter was compared to the one presented two letters back. In each session, there were 720 total trials and each had a 50% chance of being a “match.” Each stimulus (i.e., letter) was presented for 600 ms with 850 ms between stimuli.

Stroop: The Stroop task [34] is widely considered to be a measure of inhibitory control [3]. In the Stroop task, stimuli consist of a word for a color (e.g., “RED”) and a font color (e.g., green), which may or may not match the word. Individuals are asked to press a key indicating the color of the word, not the font color. This task involves inhibiting response to irrelevant and inconsistent stimuli (e.g., the word) and focusing attention on relevant stimuli (e.g., the font color). In this study, three colors were used (green, red, and blue). Three trial types were used: congruent, incongruent, and neutral. In congruent trials, red and/or green words and font color matched. In incongruent trials, red and/or green words and font color did not match. In neutral trials with red or green words and blue font color, participants did not need to respond. Stimuli were presented randomly within each trial type. In each session, there were 112 trials of each condition. The duration of each stimulus was 200 ms with 2000–2400 ms in between stimuli. To ascertain accuracy, the number of correct, incorrect, and no response occurrences for each trial type were averaged across trials; reaction time was averaged per response and trial type.

Flanker: The Flanker task is based on the Erikson paradigm [35], another common measure of inhibitory control [3]. This task typically consists of a right-facing or left-facing arrow that is immediately surrounded by arrows that all face the same direction (e.g., all facing right or all facing left). Individuals are to press a key indicating the direction of the target arrow. Two trial types were used. In congruent trials, the surrounding arrows faced the same direction as the target arrow, while in incongruent trials, the surrounding arrows faced the opposite direction. Each session consisted of 315 trials of each trial type. Stimulus duration was 150 ms.

Two measures were collected for each cognitive task. To ascertain accuracy, the number of correct, incorrect, and no response occurrences for each trial type (e.g., incongruent correct vs. incongruent incorrect vs. incongruent no response) were averaged across trials. In addition, reaction time (in milliseconds) was also averaged per response and trial type [36,37].

#### 2.3.2. Brain Activity

EEG measurement and preprocessing: As typically done [38], EEG measurement was performed during the cognitive tasks to capture electrical activity in response to cognitive stimuli. EEG data were collected at each visit for five total visits. EEG data were sampled at 300 Hz at Fz, Cz, and Pz channels using a Wearable Sensing DSI-24 dry electrode system [39,40]. Fz, Cz, and Pz were chosen prior to analysis because the frontal/central midline is the most commonly focused brain region with regards to error-related negativity [41,42]. Each trial was separated and categorized by condition (e.g., congruent or incongruent) and by response accuracy (e.g., correct or incorrect). In order to obtain enough trial data for statistical analyses, we combined condition types to focus on correct vs. incorrect responses. Individual trials were extracted −300 s to 1000 ms relative to response onset. Each trial subtracted the pre-response baseline EEG signal voltage, averaged from −300 ms to 0 ms before the response occurred. EEG measurements included error-related negativity (ERN) and power spectra, which are considered markers of executive functioning [23,43,44,45].

Standard preprocessing were run on Neuropype (neuropype.io), an EEG/biosignal data processing and collection platform by Intheon, to clean data and remove artifacts (e.g., blinks, movement). The details of the preprocessing chain are published elsewhere [46] but are presented here in brief: high-pass filter at 0.5 Hz, removal of bad channels using neighboring channel correlation and high-frequency noise detection, removal of high-artifact time windows using Artifact Subspace Reconstruction with an artifact threshold of 10 s.d., low-pass filter at 45 Hz, channel re-interpolation, and common average re-referencing. The Fz, Cz, and Pz channels analyzed in this study had adequate data quality and showed little evidence of residual blink artifacts; however, the data showed a moderate amount of noise overall, likely due to the recording environment in the mobile vehicle, reducing the sample size and statistical power of some EEG analyses. The analyses reported below only contain EEG data that had adequate data quality (i.e., absence of blink artifacts).

ERN: The ERN is a negative deflection occurring around 100 ms after response [47]. Increased (i.e., more negative) magnitude of ERN amplitudes after errors are thought to constitute better executive functioning in the form of cognitive control and monitoring and cognitive flexibility [23]. To avoid detection of inaccurate peak amplitudes [48], a wavelet-based peak finding method was applied to the session mean ERN waveform within 0 to 300 ms relative to response onset. The ERN peak amplitude and latency values were identified.

Power Spectra and Frequency Bands: The Multitaper method was used to estimate power spectral density for each trial from 0 to 500 ms relative to response onset. The PSD was normalized to account for 1/frequency fall-off and converted to decibels (dB). The normalized PSD was further averaged within frequency bands to yield bandpower estimates for the following frequency bands: delta: 1–3 Hz, theta: 4–7 Hz, alpha: 8–12 Hz, beta: 13–32 Hz, and gamma: 33–50 Hz. Increased power, especially alpha and beta power, are linked to better cognitive control and attention [49,50,51].

### 2.4. Statistical Analysis

Author G.H. from Intheon (a company, known also as intheon.io) analyzed the data using Intheon’s Neuroscale Insights biosignal data analytics platform (neuroscale.io) and was blinded to condition. For cognitive tasks, 2-factor mixed effects ANOVA models were conducted with condition, session number, with the condition and session interaction as main effects and the cognitive task reaction time or accuracy as the dependent variable. The False Discovery Rate (FDR) method [52] corrected for multiple comparisons. Multiple comparison corrections treated each electrode (Fz, Cz, Pz) as independent. For EEG analyses, linear mixed models were used since they are robust to missing data at random [53]. Each univariate EEG feature was the dependent variable, with fixed effects of condition (between-subjects factor), session number (within-subject factor), and the interaction of condition and session. A random slope was included for each participant. A mass-univariate approach, as typical for analyzing ERNs, was used to examine the effects of each factor on each EEG feature (e.g., ERN amplitude for a given channel at a specific session, or power spectral density at a specific frequency and/or session) independently. The FDR method corrected for multiple comparisons across features for each EEG channel, and not across EEG channels. Weight loss was analyzed using paired-sample t-tests to compare weight at baseline and from visit 5 (week 16). An alpha of 0.05 was used for all analyses.

## 3. Results

### 3.1. Baseline Characteristics and Weight Loss

The intervention and control conditions did not significantly differ on any of the baseline characteristics of gender, age, baseline BMI (weight and height), ethnicity, educational status, and employment status (see Table 1). Compared to baseline, the intervention group lost 7.1 pounds (SD = 9.4; −3.6% body weight) at 4 months, while the control group gained 1.0 pounds (SD = 17.5; +0.43% body weight) (t(45) = −2.03, *p* = 0.05).

### 3.2. Cognitive Tasks

#### 3.2.1. 2-Back

There were no significant differences for condition or interactions of condition and session for both accuracy and reaction time. There were significant main effects of session for correct matches (beta(5, 211) = 4.91, t = 8.45, *p* < 0.001) and correct rejections of non–matches (beta(5, 211) = 5.04, t = 8.70, *p* < 0.001) in which accuracy improved over time for the whole sample. No responses also decreased over time for both match and non-match trials (beta(5, 211) = −4.28, t = −6.14, *p* < 0.001; beta(5, 211) = −4.51, t = −6.45, *p* < 0.001). It should be noted that accuracy was generally poor on this task with many non-responses but is reported here for comprehensiveness.

#### 3.2.2. Stroop

**Figure 2:**
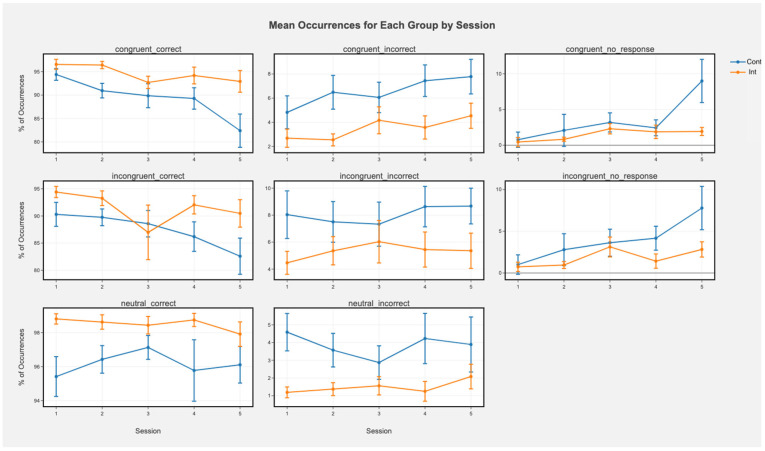
Mean occurrences (%) of each trial type for each group by session in the Stroop task. Greater % of correct trials means greater accuracy, while greater % of incorrect means lower accuracy (more errors). Error bars represent +/− SEM. Cont represents the control condition and Int represents the intervention condition.

Accuracy. There was no main effect of condition (all *p*s > 0.12). There was a main effect of session in which accuracy decreased over time for congruent incorrect (t = 3.23, *p* = 0.001), congruent correct (t = −5.41, *p* < 0.001), and incongruent correct (beta(5, 148) = 0.66, t = −3.30, *p* < 0.001) trials. This was qualified by a significant group by session interaction for congruent correct trials (Figure 2; beta(5, 148) = 1.56, t = 2.53, *p* = 0.01) in which the control group decreased in accuracy while the intervention group sustained accuracy. There were also significant main effects of session for congruent no response (beta(5, 148) = 1.71, t = 4.59, *p* < 0.001) and incongruent no response (beta(5, 148) = 1.61, t = −4.28, *p* < 0.001) trials suggesting increased no response over time. Significant group by session interactions for congruent (beta(5, 148) = −1.30, t = −2.47, *p* = 0.01) and incongruent (beta(5, 148) = −1.12, t = −2.10, *p* = 0.03) no response trials suggested that this main effect was driven primarily by an increase in no response from the control group.

Reaction time. For reaction times, there was a significant main effect of condition for incongruent correct (beta(5, 148) = 123.79, t = 3.01, *p* = 0.003) and congruent correct (beta(5, 148) = 105.73, t = 2.82, *p* = 0.005) trials in which the intervention group had faster reaction times than the control group. These were qualified by significant interaction effects for both congruent (beta(5, 148) = −17.06, t = −2.85, *p* = 0.004) and incongruent correct (beta(5, 148) = −19.20, t = −3.30, *p* < 0.001) trials in which the intervention group showed faster reaction times over time while the control group did not change over time. There were no significant main effects for the sessions (all *p*s > 0.11).

#### 3.2.3. Flanker

**Figure 3:**
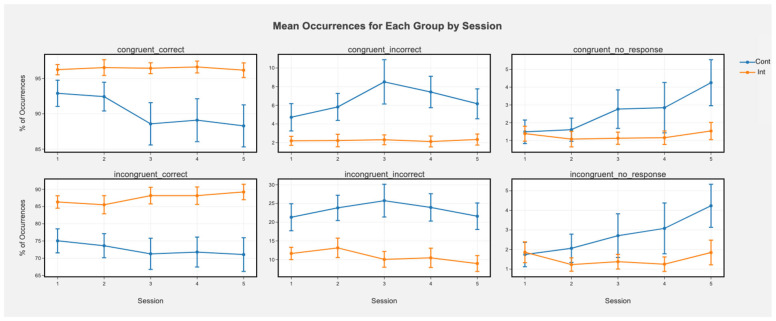
Mean occurrences (%) of each trial type for each group by session in the Flanker task. Greater % of correct trials means greater accuracy, while greater % of incorrect means lower accuracy (more errors). Error bars represent +/− SEM. Error bars represent +/− SEM. Cont represents the control condition and Int represents the intervention condition.

Accuracy. There was a main effect of session indicating that for the entire sample, accuracy decreased over time in congruent correct (beta(5, 213) = −1.46, t = −3.06, *p* < 0.001) trials and incongruent correct (beta(5, 213) = −1.18, t = −2.29, *p* = 0.03) trials. There was also a main effect of condition in which the control condition showed lower accuracy than the intervention condition for incongruent incorrect trials (beta(5, 213) = −9.60, t = −2.45, *p* = 0.01) and incongruent correct trials (beta(5, 213) = 9.39, t = 2.14, *p* = 0.03). The interactions suggest that this was driven primarily by sustained or increased accuracy over time in the intervention group but declining accuracy in the control group (Figure 3; incongruent correct: beta(5, 213) = 1.59, t = 2.19, *p* = 0.03; congruent correct: beta(5, 213) = 1.13, t = 1.67, *p* = 0.09; Figure 2). There were also main effects of session for congruent (beta(5, 213) = 1.00, t = 2.61, *p* = 0.009) and incongruent no response (beta(5, 213) = 0.94, t = 2.43, *p* = 0.01) trials in which no responses increased over time for the whole sample. Main effects of condition for no response trials were not significant (all *p*s > 0.91). There were trending, nonsignificant interactions in which the control group showed more no response over time and the interaction group was steady (congruent no response: beta(5, 213) = −0.84, t = −1.55, *p* = 0.12; incongruent no response: beta(5, 213) = −0.77, t = −1.41, *p* = 0.16).

Reaction time. There was a marginally significant main effect of session for congruent correct (beta(5, 213) = −5.27, t = −1.81, *p* = 0.07) trials, with a significant effect for incongruent correct trials (beta(5, 213) = −9.57, t = −3.12, *p* = 0.002), indicating that reaction times decreased over time for the entire sample. There was also a marginally significant main effect of condition for congruent incorrect (beta(5, 213) = 79.94, t = 1.66, *p* = 0.10) and incongruent correct trials (beta(5, 213) = 53.03, t = 1.93, *p* = 0.053) in which the intervention condition had faster reaction times than the control condition across trials. There were marginally significant interactions in which the control condition showed slower reaction time over time and the intervention condition was stable for congruent correct (beta(5, 213) = 8.22, t = 1.93, *p* = 0.053) and incongruent incorrect (beta(5, 213) = 9.21, t = 1.61, *p* = 0.11) sessions.

### 3.3. Brain Activity

**Figure 4:**
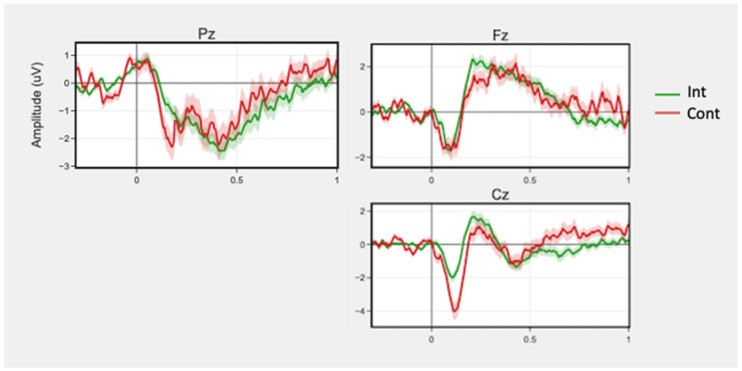
Mean ERN waveforms for correct and incorrect trials across sessions by group for channels Fz, Pz, and Cz. Time 0 represents response onset. Shaded regions around the lines represent +/− SEM.

ERN waveforms are displayed in Figure 4.

#### 3.3.1. 2-Back

EEG results were unable to be analyzed due to the overall poor performance (particularly in the large number of no responses) across most subjects on the task. There were too few correct and incorrect trials to calculate and measure the ERN response, which is based on incorrect responses.

#### 3.3.2. Stroop

There was no significant main effect of condition for ERN peak amplitude or latency. There was a marginally significant condition by session interaction (beta(5, 148) = −0.55, t = −1.76, *p* = 0.08; beta(5, 148) = 0.01, t = 2.00, *p* = 0.08, respectively) for incorrect trials in peak amplitude in the Fz region. There was a significant main effect for condition for Pz beta (beta(5, 148) = 2.34, t = 2.51, *p* = 0.03) and gamma (beta(5, 148) = 3.43, t = 2.97, *p* = 0.01) band powers for incorrect trials. The intervention group showed increased power compared to the control group. No interactions of condition and session were significant.

#### 3.3.3. Flanker

There were no significant main effects of condition for peak amplitude and latency. There was greater ERN magnitude (i.e., more negative peak amplitude) in the Cz region during incorrect responses in the intervention group compared to the control (beta(5, 213) = −1.96, t = −1.36, *p* = 0.35), but this was not significant with FDR adjustment.

## 4. Discussion

There is suggestive, but generally mixed, evidence on whether generalized interactive weight management interventions improve executive function; improvements have been found in some studies but not others [6,12,13]. Further, it is unknown whether a CBT-based generalized weight management intervention would show an impact on executive functioning. This pilot RCT provides preliminary evidence that this mobile CBT-based intervention helped to maintain or improve executive function over time compared to a control group. Specifically, the intervention group showed better sustained performance on executive function-related cognitive tasks compared to a control group, which showed decreased or no improvement in performance over four months. Further, the intervention group showed a few trends of greater executive function-related brain activity. Finally, the intervention group lost significantly more weight at 16 weeks than the control group. Overall, these results are in line with other mindfulness interventions in other contexts (i.e., not weight management), as well as weight interventions such as CRT-O that focused primarily on cognitive training and some standard generalized weight interventions [11,12,14,18,20,21]. Our results also corroborate some previous work on cognitive training plus weight management interventions showing stable or improved self-reported executive functioning in the treatment group and declining self-reported executive functioning in the usual care group, though these studies did not find differences in weight loss [54,55]. Our findings, at the very least, provide pilot, randomized prospective data that an interactive CBT-based weight management program showed executive functioning improvements compared to weekly weight-related newsletters, which is important given the need for more data from weight management interventions, particularly CBT-based ones. While we speculate that these results are due to the CBT program components rather than engagement in weight loss practices (e.g., physical activity or healthy eating) given theoretical and empirical links between CBT (including third-wave CBT such as mindfulness) and executive functioning [56,57], due to other differences between the intervention and control, future research is required to confirm this speculation; possible designs are discussed in more detail below.

### 4.1. Cognitive Tasks and Brain Activity

We found that the intervention group had higher accuracy overall and over time in the Flanker test for incongruent trials, which tend to involve inhibitory control and conflict monitoring processes. Inspection of the plots suggests that the control group decreased in accuracy over time and the intervention group was able to sustain accuracy. These results suggest that the intervention enabled sustained inhibitory control over time, in contrast to declines in the control group. Similarly, in the Stroop task measuring inhibitory control and conflict monitoring, the intervention group maintained accuracy in congruent correct trials while the control group decreased in accuracy. The control group also failed to provide a response increasingly over time while the intervention group remained consistent. The intervention group also showed faster reaction times than the control group for congruent and incongruent correct trials. The intervention group showed faster reaction times over time while the control group did not. Thus, in both the Flanker and Stroop tasks, the intervention group had better performance over time in congruent correct responses. Taken together, these results suggest that the intervention helped individuals to maintain inhibitory control in comparison to the control group which showed declines over time in accuracy and reaction time.

To our knowledge, it is unknown to what extent individuals with obesity sustain performance on multiple, separated cognitive tasks over time periods as long as a month. We speculate that mental fatigue may explain the control condition’s decline in performance over time for the Flanker and Stroop tasks. There is evidence suggestive that obesity could be related to greater experienced and perceived mental fatigue [58,59,60,61,62]. In the current study, the 2-back task was always completed first, followed by the Stroop and Flanker tasks. The 2-back task is the most difficult of the cognitive tasks in this study and has been shown to induce mental fatigue and declines in performance [63]. Despite the task’s difficulty, both conditions were able to improve their performance over time. Thus, it is possible that, though this fatiguing task was presented first in order to mimic real-life conditions during weight management in which executive functioning may become depleted over multiple food choice or other decisions as opposed to a relatively fresh mind in a laboratory setting, the attention required to perform increasingly better on this task may have led to greater fatigue on subsequent tasks and therefore declining performance (Stroop and Flanker) in individuals with obesity without cognitive intervention, as in the control group. In contrast, perhaps CBT and mindfulness helped to sustain the intervention condition’s attention and perseverance. There are other possible mechanisms as well. There was a difference in weight loss between the groups in which the intervention group lost significantly more weight than the control group, and research suggests that weight loss may alleviate health conditions or hormonal dysregulations associated with detriments in executive function [64]. It is thus possible that the greater weight loss in the intervention group resulted in more sustained executive functioning over time compared to the control group. Another potential mechanism is differential motivation. It is possible that the intervention group, which lost weight over time, was more motivated to pay closer attention to repeating difficult tasks in this weight loss study compared to the control group who did not experience weight loss success. Future studies should test these potential explanations.

We did not find group differences or interactions for the 2-back test. This could be because the 2-back is a very difficult cognitive task and accuracy was poor overall for this task; this may have reduced differences between the groups. Another potential explanation is that the 2-back test measures working memory while the Flanker and Stroop task are more associated with inhibitory control and conflict monitoring. It may be possible that CBT-based interventions do not affect working memory as much as inhibitory control, as a meta-analysis found that mindfulness interventions improved executive functions like inhibitory control but not working memory [56], and another study found that a CBT intervention led to improvements primarily in inhibitory control (i.e., Stroop performance) but not other components of executive function [65]. Future studies should examine this further.

Overall, these results align with previous studies of CBT and mindfulness interventions in other domains that have shown improvements in higher-order executive functions such as inhibitory control as measured by the Stroop and Flanker tasks [56,65,66,67].

The EEG results were more marginal; future research should follow up on the effect sizes of these exploratory results in larger samples. During the Stroop test, Pz beta and gamma band powers in incorrect trials were significantly greater for the intervention group than the control group. Frontal and parietal beta power have been linked to increased cognitive control, attentiveness and attentive control, planning, and memory [45,68,69]. There was also a trend towards greater Fz ERN magnitude in the intervention group. Generally, greater ERN magnitudes (i.e., more negative peak amplitudes) are seen as reflections of better performance and conflict monitoring [23], but greater ERN magnitudes on the same task over time could be indicative of more efficient and adaptable processing [70]. In this case, ERN magnitudes in the control group did not increase, which may suggest that there was a marginal trend of more efficient processing and control of resources in the intervention group.

For the Flanker test, for which EEG data quality was noisier than the Stroop test, there were trends towards greater ERN magnitude in the Cz region, and theta band power on incorrect trials in the intervention condition. Greater ERN responses following errors is indicative of efficient cognitive information processing and control [71]. Theta band power is reflective of conflict monitoring and strategic control [72,73].

### 4.2. Limitations and Future Directions

Limitations of this study include lower signal quality in the study data compared to data quality pre-testing, most likely due to the use of the mobile testing center, which was necessitated by the start of the COVID-19 pandemic. However, this affected all sessions equally, so it did not differentially impact some tasks or sessions over others. Future studies may benefit from using a consistent test environment that has been evaluated using pilot data prior to substantial data collection, to help ensure external noise factors are adequately addressed. Another limitation is that EEG analysis could not be conducted on the 2-back task because of the prominence of no response trials, as error responses are necessary to analyze the ERN component. This could be due to the difficulty of the 2-back test as participants qualitatively reported that they found it to be exceptionally challenging. Moreover, this study also had a small overall sample size with high initial dropout after randomization, most likely due to the start of the COVID-19 pandemic. As a result, there were nonsignificant trends in the data that future studies should confirm with larger sample sizes. Finally, the participants were more educated and of higher income than the general population, so future studies should determine how generalizable the results are to other populations.

Given the design of this pilot study that involved a control group that received weekly “usual care” in the form of publicly available resources, it is impossible to tell if results (executive functioning and weight loss) are solely to the CBT nature of the program or to the more structured weight management intervention as provided by the mobile program, or both. There are many differences between the weekly “usual care” and the intervention aside from its CBT foundation, such as greater individual personalization to specific weight loss goals and interactivity because of the availability of health coaches and a peer group, which could be responsible for the results. It should be noted that the intervention did not contact participants about the availability of new daily articles, and the digital nature meant that participants had to self-manage their own participation in the absence of study participation requirements or contact typically present in structured in-person interventions [74,75]. Still, in future well-powered randomized controlled trials, we plan to directly compare the effects of the program compared to a similar experience without CBT and to no intervention. Future studies also will aim to explore the relationship between weight loss and executive functioning over time in order to ascertain to what extent weight loss itself, aside from cognitive aspects of the program, directly relates to improved executive functioning, or vice versa. Future studies can use frequent repeated measurements of weight and executive functioning over time to model the predictive relationships at hand using longitudinal models. Moreover, given the importance of understanding weight maintenance and not just short-term weight loss and initial evidence of differential decision making in those who maintain their weight loss [6,76], future studies will evaluate executive functioning in those who successfully maintained compared to those who did not maintain their weight long-term.

## 5. Conclusions

A structured CBT-based generalized weight management intervention resulted in sustained executive functioning compared to a control group receiving weekly weight loss resources. Specifically, the intervention group showed evidence of increased inhibitory control and performance monitoring and, to a lesser extent, executive function-related brain activity. Our results contribute data to the mixed and limited literature on weight management interventions, and in particular CBT weight interventions, and pre-post changes in executive function or brain activity. Future studies should examine what mediates these improvements, as well as explore if our results generalize to other populations or CBT-based behavior change interventions.

## Figures and Tables

**Table 1:** Demographics and baseline characteristics.

	Intervention	Control	*p*-Value
**Gender**			0.99
Female	22 (88%)	20 (83.3%)	
Male	3 (12%)	4 (16.7%)	
**Age**	46.3	42.1	0.09
**Baseline BMI**	33.0	36.1	0.20
**Ethnicity**			0.30
Asian or Pacific Islander	3 (12%)	0	
Black/African	3 (12%)	1 (4.2%)	
Caucasian	15 (60%)	19 (79.2%)	
Hispanic/Latino	2 (8%)	3 (12.5%)	
Other	2 (8%)	1 (4.2%)	
**Education**			0.50
High school degree	1 (4%)	2 (8.3%)	
Some college	4 (16%)	3 (12.5%)	
2-year college degree or vocational training	1 (4%)	4 (16.7%)	
4-year college degree	9 (36%)	5 (20.8%)	
Graduate degree (Master’s, PhD, MD, JD)	10 (40%)	10 (41.7%)	
**Employment status**			0.20
Full-time employed	17 (68%)	21 (87.5%)	
Part-time employed	5 (20%)	0 (0%)	
Self-employed	1 (4%)	1 (4.2%)	
Unemployed	0 (0%)	1 (4.2%)	
Looking after family	2 (8%)	1 (4.2%)	

## Data Availability

Restrictions apply to the availability of these data. Data were obtained from Noom and are available by request from the corresponding author with the permission of Noom.

## References

[1] Blüher M. (2019). Obesity: Global Epidemiology and Pathogenesis. Nat. Rev. Endocrinol..

[2] Diabetes Prevention Program (DPP) Research Group (2002). The Diabetes Prevention Program (DPP): Description of Lifestyle Intervention. Diabetes Care.

[3] Favieri F., Forte G., Casagrande M. (2019). The Executive Functions in Overweight and Obesity: A Systematic Review of Neuropsychological Cross-Sectional and Longitudinal Studies. Front. Psychol..

[4] Elliott R. (2003). Executive Functions and Their Disorders: Imaging in Clinical Neuroscience. Br. Med. Bull..

[5] Banich M.T. (2009). Executive Function: The Search for an Integrated Account. Curr. Dir. Psychol. Sci..

[6] Gettens K.M., Gorin A.A. (2017). Executive Function in Weight Loss and Weight Loss Maintenance: A Conceptual Review and Novel Neuropsychological Model of Weight Control. J. Behav. Med..

[7] Spitznagel M.B., Alosco M., Strain G., Devlin M., Cohen R., Paul R., Crosby R.D., Mitchell J.E., Gunstad J. (2013). Cognitive Function Predicts 24-Month Weight Loss Success after Bariatric Surgery. Surg. Obes. Relat. Dis..

[8] Butryn M.L., Martinelli M.K., Remmert J.E., Roberts S.R., Zhang F., Forman E.M., Manasse S.M. (2019). Executive Functioning as a Predictor of Weight Loss and Physical Activity Outcomes. Ann. Behav. Med..

[9] Best J.R., Nagamatsu L.S., Liu-Ambrose T. (2014). Improvements to Executive Function during Exercise Training Predict Maintenance of Physical Activity over the Following Year. Front. Hum. Neurosci..

[10] Daly M., McMinn D., Allan J.L. (2014). A Bidirectional Relationship between Physical Activity and Executive Function in Older Adults. Front. Hum. Neurosci..

[11] Raman J., Hay P., Tchanturia K., Smith E. (2018). A Randomised Controlled Trial of Manualized Cognitive Remediation Therapy in Adult Obesity. Appetite.

[12] Veronese N., Facchini S., Stubbs B., Luchini C., Solmi M., Manzato E., Sergi G., Maggi S., Cosco T., Fontana L. (2017). Weight Loss Is Associated with Improvements in Cognitive Function among Overweight and Obese People: A Systematic Review and Meta-Analysis. Neurosci. Biobehav. Rev..

[13] Smith E., Hay P., Campbell L., Trollor J.N. (2011). A Review of the Association between Obesity and Cognitive Function across the Lifespan: Implications for Novel Approaches to Prevention and Treatment. Obes. Rev..

[14] Allom V., Mullan B., Smith E., Hay P., Raman J. (2018). Breaking Bad Habits by Improving Executive Function in Individuals with Obesity. BMC Public Health.

[15] Castelnuovo G., Pietrabissa G., Manzoni G.M., Cattivelli R., Rossi A., Novelli M., Varallo G., Molinari E. (2017). Cognitive Behavioral Therapy to Aid Weight Loss in Obese Patients: Current Perspectives. Pseudo Rigid Body Model.

[16] Comsa L., David O., David D. (2020). Outcomes and Mechanisms of Change in Cognitive-Behavioral Interventions for Weight Loss: A Meta-Analysis of Randomized Clinical Trials. Behav. Res. Ther..

[17] Cuijpers P., van Straten A., Andersson G. (2008). Internet-Administered Cognitive Behavior Therapy for Health Problems: A Systematic Review. J. Behav. Med..

[18] Chiesa A., Calati R., Serretti A. (2011). Does Mindfulness Training Improve Cognitive Abilities? A Systematic Review of Neuropsychological Findings. Clin. Psychol. Rev..

[19] Chow G., Gan J.K.E., Chan J.K.Y., Wu X.V., Klainin-Yobas P. (2021). Effectiveness of Psychosocial Interventions among Older Adults with Mild Cognitive Impairment: A Systematic Review and Meta-Analysis. Aging Ment. Health.

[20] Leyland A., Rowse G., Emerson L.-M. (2019). Experimental Effects of Mindfulness Inductions on Self-Regulation: Systematic Review and Meta-Analysis. Emotion.

[21] Pozuelos J., Mead B., Rueda M., Malinowski P. (2019). Short-Term Mindful Breath Awareness Training Improves Inhibitory Control and Response Monitoring. Prog. Brain Res..

[22] Brouwer A.-M., Hogervorst M.A., van Erp J.B.F., Heffelaar T., Zimmerman P.H., Oostenveld R. (2012). Estimating Workload Using EEG Spectral Power and ERPs in the N-Back Task. J. Neural. Eng..

[23] Larson M.J., Clayson P.E. (2011). The Relationship between Cognitive Performance and Electrophysiological Indices of Performance Monitoring. Cogn. Affect. Behav. Neurosci..

[24] Kim N.Y., Wittenberg E., Nam C.S. (2017). Behavioral and Neural Correlates of Executive Function: Interplay between Inhibition and Updating Processes. Front. Neurosci..

[25] Fissler M., Winnebeck E., Schroeter T.A., Gummbersbach M., Huntenburg J.M., Gärtner M., Barnhofer T. (2017). Brief Training in Mindfulness May Normalize a Blunted Error-Related Negativity in Chronically Depressed Patients. Cogn. Affect. Behav. Neurosci..

[26] Lomas T., Ivtzan I., Fu C.H.Y. (2015). A Systematic Review of the Neurophysiology of Mindfulness on EEG Oscillations. Neurosci. Biobehav. Rev..

[27] Crook T.H., Lebowitz B.D., Pirozzolo F.J., Zappalà G., Cavarzeran F., Measso G., Massari D.C. (1993). Recalling Names after Introduction: Changes across the Adult Life Span in Two Cultures. Dev. Neuropsychol..

[28] Lande M.B., Kaczorowski J.M., Auinger P., Schwartz G.J., Weitzman M. (2003). Elevated Blood Pressure and Decreased Cognitive Function among School-Age Children and Adolescents in the United States. J. Pediatr..

[29] Stubbs J., Whybrow S., Teixeira P., Blundell J., Lawton C., Westenhoefer J., Engel D., Shepherd R., Mcconnon Á., Gilbert P. (2011). Problems in Identifying Predictors and Correlates of Weight Loss and Maintenance: Implications for Weight Control Therapies Based on Behaviour Change: Predicting Weight Outcomes. Obes. Rev..

[30] Hartmann-Boyce J., Boylan A.-M., Jebb S.A., Fletcher B., Aveyard P. (2017). Cognitive and Behavioural Strategies for Self-Directed Weight Loss: Systematic Review of Qualitative Studies. Obes. Rev..

[31] Krueger C., Tian L. (2004). A Comparison of the General Linear Mixed Model and Repeated Measures ANOVA Using a Dataset with Multiple Missing Data Points. Biol. Res. Nurs..

[32] Kirchner W.K. (1958). Age Differences in Short-Term Retention of Rapidly Changing Information. J. Exp. Psychol..

[33] Lamichhane B., Westbrook A., Cole M.W., Braver T.S. (2020). Exploring Brain-Behavior Relationships in the N-Back Task. NeuroImage.

[34] Stroop J.R. (1935). Studies of Interference in Serial Verbal Reactions. J. Exp. Psychol..

[35] Eriksen C.W., Schultz D.W. (1979). Information Processing in Visual Search: A Continuous Flow Conception and Experimental Results. Percept. Psychophys..

[36] McDermott J.M., Pérez-Edgar K., Fox N.A. (2007). Variations of the Flanker Paradigm: Assessing Selective Attention in Young Children. Behav. Res..

[37] Bugg J.M., Jacoby L.L., Toth J.P. (2008). Multiple Levels of Control in the Stroop Task. Mem. Cogn..

[38] Gevins A.S., Zeitlin G.M., Yingling C.D., Doyle J.C., Dedon M.F., Schaffer R.E., Roumasset J.T., Yeager C.L. (1979). EEG Patterns during ‘Cognitive’ Tasks. I. Methodology and Analysis of Complex Behaviors. Electroencephalogr. Clin. Neurophysiol..

[39] Slater J.D., Kalamangalam G.P., Hope O. (2012). Quality Assessment of Electroencephalography Obtained from a “Dry Electrode” System. J. Neurosci. Methods.

[40] Halford J.J., Schalkoff R.J., Satterfield K.E., Martz G.U., Kutluay E., Waters C.G., Dean B.C. (2016). Comparison of a Novel Dry Electrode Headset to Standard Routine EEG in Veterans. J. Clin. Neurophysiol..

[41] Debener S., Ullsperger M., Siegel M., Fiehler K., Von Cramon D.Y., Engel A.K. (2005). Trial-by-Trial Coupling of Concurrent Electroencephalogram and Functional Magnetic Resonance Imaging Identifies the Dynamics of Performance Monitoring. J. Neurosci..

[42] Maier M.E., di Pellegrino G., Steinhauser M. (2012). Enhanced Error-Related Negativity on Flanker Errors: Error Expectancy or Error Significance?. Psychophysiology.

[43] Yasuda A., Sato A., Miyawaki K., Kumano H., Kuboki T. (2004). Error-Related Negativity Reflects Detection of Negative Reward Prediction Error. NeuroReport.

[44] Folstein J.R., Van Petten C. (2008). Influence of Cognitive Control and Mismatch on the N2 Component of the ERP: A Review. Psychophysiology.

[45] Tafuro A., Ambrosini E., Puccioni O., Vallesi A. (2019). Brain Oscillations in Cognitive Control: A Cross-Sectional Study with a Spatial Stroop Task. Neuropsychologia.

[46] Mullen T.R., Kothe C.A., Chi Y.M., Ojeda A., Kerth T., Makeig S., Jung T.-P., Cauwenberghs G. (2015). Real-Time Neuroimaging and Cognitive Monitoring Using Wearable Dry EEG. IEEE Trans. Biomed. Eng..

[47] Gehring W.J., Goss B., Coles M.G., Meyer D.E., Donchin E. (1993). A Neural System for Error Detection and Compensation. Psychol. Sci..

[48] Řondík T., Ciniburk J., Mouček R., Mautner P. ERP Components Detection Using Wavelet Transform and Matching Pursuit Algorithm. Proceedings of the International Conference on Applied Electronics.

[49] Ambrosini E., Vallesi A. (2016). Asymmetry in Prefrontal Resting-State EEG Spectral Power Underlies Individual Differences in Phasic and Sustained Cognitive Control. Neuroimage.

[50] Compton R.J., Arnstein D., Freedman G., Dainer-Best J., Liss A. (2011). Cognitive Control in the Intertrial Interval: Evidence from EEG Alpha Power: Cognitive Control in the Intertrial Interval. Psychophysiology.

[51] Friese U., Daume J., Göschl F., König P., Wang P., Engel A.K. (2016). Oscillatory Brain Activity during Multisensory Attention Reflects Activation, Disinhibition, and Cognitive Control. Sci. Rep..

[52] Benjamini Y., Hochberg Y. (1995). Controlling the False Discovery Rate: A Practical and Powerful Approach to Multiple Testing. J. R. Stat. Soc. Ser. B. Methodol..

[53] Schielzeth H., Dingemanse N.J., Nakagawa S., Westneat D.F., Allegue H., Teplitsky C., Réale D., Dochtermann N.A., Garamszegi L.Z., Araya-Ajoy Y.G. (2020). Robustness of Linear Mixed-Effects Models to Violations of Distributional Assumptions. Methods Ecol. Evol..

[54] Verbeken S., Braet C., Goossens L., van der Oord S. (2013). Executive Function Training with Game Elements for Obese Children: A Novel Treatment to Enhance Self-Regulatory Abilities for Weight-Control. Behav. Res. Ther..

[55] Verbeken S., Braet C., Naets T., Houben K., Boendermaker W., Zeepreventorium V.Z.W. (2018). Computer Training of Attention and Inhibition for Youngsters with Obesity: A Pilot Study. Appetite.

[56] Im S., Stavas J., Lee J., Mir Z., Hazlett-Stevens H., Caplovitz G. (2021). Does Mindfulness-Based Intervention Improve Cognitive Function?: A Meta-Analysis of Controlled Studies. Clin. Psychol. Rev..

[57] Gilbert M., Raman J., Sui Z. (2021). Cognitive Remediation-Enabled Cognitive Behaviour Therapy for Obesity: A Case Series. Eat. Weight Disord..

[58] Duan X., Rhee J., Mehta R.K., Srinivasan D. (2018). Neuromuscular Control and Performance Differences Associated With Gender and Obesity in Fatiguing Tasks Performed by Older Adults. Front. Physiol..

[59] Gholami Z., Akhlaghi M. (2021). The Effect of Flaxseed on Physical and Mental Fatigue in Children and Adolescents with Overweight/Obesity: A Randomised Controlled Trial. Br. J. Nutr..

[60] Gletsu-Miller N., Shevni N., Manatunga A., Lin E., Musselman D. (2019). A Multidimensional Analysis of the Longitudinal Effects of Roux En y Gastric Bypass on Fatigue: An Association with Visceral Obesity. Physiol. Behav..

[61] Cserjési R., Luminet O., Poncelet A.-S., Lénárd L. (2009). Altered Executive Function in Obesity. Exploration of the Role of Affective States on Cognitive Abilities. Appetite.

[62] Mamrot P., Hanć T. (2019). The Association of the Executive Functions with Overweight and Obesity Indicators in Children and Adolescents: A Literature Review. Neurosci. Biobehav. Rev..

[63] Tanaka M., Shigihara Y., Ishii A., Funakura M., Kanai E., Watanabe Y. (2012). Effect of Mental Fatigue on the Central Nervous System: An Electroencephalography Study. Behav. Brain Funct..

[64] Spitznagel M.B., Hawkins M., Alosco M., Galioto R., Garcia S., Miller L., Gunstad J. (2015). Neurocognitive Effects of Obesity and Bariatric Surgery. Eur. Eat. Disord. Rev..

[65] Delgado-Rico E., Río-Valle J.S., Albein-Urios N., Caracuel A., González-Jiménez E., Piqueras M.J., Brandi P., Ruiz-López I.M., García-Rodríguez I., Martín-Matillas M. (2012). Effects of a Multicomponent Behavioral Intervention on Impulsivity and Cognitive Deficits in Adolescents with Excess Weight. Behav. Pharmacol..

[66] Norris C.J., Creem D., Hendler R., Kober H. (2018). Brief Mindfulness Meditation Improves Attention in Novices: Evidence From ERPs and Moderation by Neuroticism. Front. Hum. Neurosci..

[67] Wang Y., Xin T.-T., Liu X.-H., Zhang Y., Lu H.-H., Zhai Y.-B. (2012). Mindfulness Can Reduce Automatic Responding: Evidences from Stroop Task and Prospective Memory Task. Acta Psychol. Sin..

[68] Stoll F., Wilson C., Faraut M., Vezoli J., Knoblauch K., Procyk E. (2016). The Effects of Cognitive Control and Time on Frontal Beta Oscillations. Cereb. Cortex.

[69] Herrmann C.S., Strüber D., Helfrich R.F., Engel A.K. (2016). EEG Oscillations: From Correlation to Causality. Int. J. Psychophysiol..

[70] Riesel A., Kathmann N., Klawohn J. (2019). Flexibility of Error-Monitoring in Obsessive-Compulsive Disorder under Speed and Accuracy Instructions. J. Abnorm. Psychol..

[71] Holroyd C.B., Coles M.G.H. (2002). The Neural Basis of Human Error Processing: Reinforcement Learning, Dopamine, and the Error-Related Negativity. Psychol. Rev..

[72] Cavanagh J.F., Figueroa C.M., Cohen M.X., Frank M.J. (2012). Frontal Theta Reflects Uncertainty and Unexpectedness during Exploration and Exploitation. Cereb. Cortex.

[73] Viviani G., Vallesi A. (2021). EEG-Neurofeedback and Executive Function Enhancement in Healthy Adults: A Systematic Review. Psychophysiology.

[74] Santarossa S., Kane D., Senn C.Y., Woodruff S.J. (2018). Exploring the Role of In-Person Components for Online Health Behavior Change Interventions: Can a Digital Person-to-Person Component Suffice?. J. Med. Internet Res..

[75] Muñoz R.F., Leykin Y., Barrera A.Z., Brown C.H., Bunge E.L. (2017). The Impact of Phone Calls on Follow-up Rates in an Online Depression Prevention Study. Internet Interv..

[76] Gowey M.A., Neumeier W.H., Henry S., Wadley V.G., Phillips J., Hayden K.M., Espeland M.A., Coday M., Lewis C.E., Dutton G.R. (2021). Executive Function in Individuals with Clinically Significant Weight Loss via Behavioral Intervention. Obes. Sci. Pract..

